# Weight Loss After Bariatric Surgery in Morbidly Obese End-Stage Kidney Disease Patients as Preparation for Kidney Transplantation. Matched Pair Analysis in a High-Volume Bariatric and Transplant Center

**DOI:** 10.1007/s11695-020-04555-8

**Published:** 2020-04-05

**Authors:** Małgorzata Dobrzycka, Monika Proczko-Stepaniak, Łukasz Kaska, Maciej Wilczyński, Alicja Dębska-Ślizień, Jarosław Kobiela

**Affiliations:** 1 Department of General, Endocrine and Transplant Surgery, Medical University of Gdansk, Gdansk, Poland; 2 Department of Nephrology and Transplant Medicine, Medical University of Gdansk, Gdansk, Poland

**Keywords:** Bariatric surgery, Kidney transplantation, End-stage kidney disease, Weight loss

## Abstract

**Background:**

The number of morbidly obese kidney transplant candidates is growing. They have limited access to kidney transplantation and are at a higher risk of postoperative complications. Bariatric surgery is considered as a safe weight loss method in those patients.

**Objectives:**

Matched pair analysis was designed to analyze the preparatory and postoperative weight loss after bariatric procedures in end-stage kidney disease (ESKD) and non-ESKD morbidly obese patients.

**Methods:**

Twenty patients with ESKD underwent bariatric surgery in our Centre of Excellence for Bariatric and Metabolic Surgery between 2015 and 2019 (nine one-anastomosis gastric bypasses, nine Roux-en-Y gastric bypasses, and two sleeve gastrectomies). They were compared with matched pairs from a dataset of 1199 morbidly obese patients without ESKD. Data on demographic factors and comorbidities was recorded. BMI was obtained at the start of the preparatory period preceding the bariatric procedure, at the time of procedure, and during the 1-year follow-up.

**Results:**

The ESKD and non-ESKD patients did not differ significantly in preoperative weight loss (13.00 ± 11.69 kg and 15.22 ± 15.96 kg respectively, *p* = 0.619). During the 1-year follow-up, the weight loss was similar to the non-ESKD group. In the first 3 months, faster weight loss in ESKD was observed. Initial and follow-up BMI values did not differ significantly between groups. We demonstrated that obese patients with ESKD can lose weight as effectively as non-ESKD patients.

**Conclusion:**

Morbidly obese ESKD patients have an equal weight loss to patients without ESKD. Bariatric surgery could improve access to kidney transplantation and may potentially improve transplantation outcomes of obese patients with ESKD.

## Introduction

Obesity is a growing problem worldwide. The prevalence of obesity has doubled since 1980, and now, nearly a third of the world’s population is classified as overweight or obese. Obesity is associated with numerous comorbidities including diabetes type 2, peripheral vascular disease, cardiovascular disease, asthma, and osteoarthritis and was shown to be an independent risk factor for developing an end-stage kidney disease (ESKD) [1, 2]. Moreover, the relative risk correlates with a higher body mass index (BMI). The risk of ESKD progression was also increased by obesity [3]. Due to the obesity epidemic, the number of obese transplant candidates has also increased. Nearly 60% of all kidney transplant recipients are overweight or obese with male predominance [4]. It was proven that obesity limits, or at least delays, access to kidney transplantation [5]. Bariatric surgery is considered a safe method to achieve rapid and sustainable weight loss before kidney transplantation [6]. Pretransplant weight loss may allow to sustain body weight and prevent common post-transplant weight gain [7]. In the current study, we compare the preoperative and postoperative weight loss in patients with ESKD with matched controls without ESKD treated with bariatric surgery in a high-volume bariatric and transplant center.

## Subjects and Methods

We have compared retrospectively the dynamics of weight loss of patients with and without ESKD who underwent bariatric surgery in a large bariatric and transplant center. In our institutional registries, 20 patients with ESKD were treated for morbid obesity between 2015 and 2019. ESKD was defined as the presence of an estimated glomerular filtration rate less than 15 mL/min/1.7 m^2^ [8].

For the purpose of this analysis, patients were matched for age, gender, and type of surgery with 20 patients without ESKD from our institutional dataset of 1199 patients. Matching was executed in a 1 to 1 ratio based on data queries. Data analysis included patient’s demographics. The ESKD patients (14 males, 6 females) were between 42 and 64 years of age. The non-ESKD group consisted of 14 males and 6 females between 42 and 64 years of age. Body weight (kg) and body height (m) were used to calculate BMI. All patients were qualified to bariatric procedure according to IFSO guidelines after a multidisciplinary consultation [9]. Patients were qualified to surgery if their BMI exceeded 40 kg/m^2^ or they had BMI between 35 and 40 kg/m^2^ with significant comorbidities. The preoperative diet plan is exercised under the supervision of a dietitian. It is designed to give approximately 100 g of carbohydrate per day, low in fat and moderate in protein. The energy value of the diet is between 800 and 1000 kcal per day. Two weeks before surgery, a liver-shrinking diet (600 kcal per day) was administered. ESKD patients had consulted with a nephrologist and undergone hemodialysis without heparin 1 day before surgery and on postoperative day 1. They do not require distinctions in anesthesia compared with the non-ESKD group. Postoperatively, routine thromboembolism prophylaxis with dalteparin sodium (5000 IU subcutaneous) was conducted except on the days with hemodialysis. The maximal BMI was calculated from maximal body weight at the start of the preparatory period for bariatric treatment. The initial BMI was calculated from the weight at the time of surgery after an obligatory weight loss period with the low-calorie diet. Three bariatric procedures were performed according to our institutional regulations by the same surgeons [10]. The follow-up measurements were performed during routine follow-up in the outpatient department 1, 3, 6, and 12 months after surgery.

Weight loss was reported according to the American Society for Metabolic and Bariatric Surgery (ASMBS) guidelines [11]. The change in BMI (ΔBMI) was calculated according to the formula = (initial BMI) − (postoperative BMI on each follow-up point). The % of reduction of BMI was calculated according to the formula = follow-up BMI/initial BMI × 100% on each follow-up point. The percentage of excess BMI loss (%EBMIL) was calculated using the formula = [ΔBMI/(Initial BMI − 25)] × 100% [11]. In the preparatory period, we used maximal BMI to calculate %EBMIL.

### Statistical Analysis

Statistical analyses were performed using the Statistica 13.3 Software (TIBCO Software Inc.). Data was presented as mean ± standard deviation. The Chi^2^ and t-tests were used for comparisons. Statistical significance was considered for *p* < 0.05. The graphs were drawn in the Microsoft Excel software.

## Results

Baseline demographic characteristics of the patients are presented in Table 1.

**Table 1:** Baseline demographic characteristics of the patients

	ESKD group (*n* = 20)	Non-ESKD group (*n* = 20)	p*
Gender (male/female)	70/30%	70/30%	n/a
Dialysis type			
Hemodialysis	16 (80%)	0	n/a
Peritoneal dialysis	2 (10%)	0	n/a
Preemptive	2 (10%)	0	n/a
ESKD etiology			
Diabetes mellitus	4	0	n/a
Hypertension	3	0	n/a
Glomerulonephritis	4	0	n/a
ADPKD	3	0	n/a
Unknown	6	0	n/a
Comorbidities			
Hypertension	14 (70%)	7 (35%)	0.027
Diabetes mellitus type 2	12 (60%)	10 (50%)	0.525
Hyperlipidemia	5 (25%)	2 (10%)	0.212
Coronary disease	6 (30%)	0	
Obstructive sleep apnea	2 (10%)	1 (5%)	0.548
Gastrointestinal reflux disease	3 (15%)	3 (15%)	1
Chronic obstructive pulmonary disease	4 (20%)	1 (5%)	0.340
Cardiac rhythm abnormalities	4 (20%)	0	
Previous surgeries	12 (60%)	10 (50%)	0.751

*Chi^2^ test was used for all comparisons

### ESKD Group

The ESKD patients’ mean maximal weight was 128 ± 18.5 kg and was reduced to 115 ± 14.5 kg preoperatively. In the preparatory period, a 10% reduction of body weight was achieved. Mean maximal BMI was 42.8 kg/m^2^; the initial BMI was 38.5 kg/m^2^. In the baseline characteristics of ESKD, a higher incidence of hypertension was noted (70% vs 35%, *p* = 0.027). OAGB (one-anastomosis gastric bypass) was performed in 9/20 patients (45%), RYGB (Roux-en-Y gastric bypass) in 9/20 patients (45%), LSG (laparoscopic sleeve gastrectomy) in 2/20 patients (10%). In the ESKD group, one serious complication on the first postoperative day after OAGB was noted. The patient required revision surgery for a leak at the gastrojejunal anastomosis, which was identified and sutured. In ESKD patients, the length of hospital stay was 3.8 ± 0.5 day (excluded one case with LOS of 26 days).

### Matched Non-ESKD Group

The non-ESKD patients’ mean maximal weight was 131.5 ± 20.5 kg and was reduced to 116.3 ± 14.5 kg preoperatively. In the control group, a 12% reduction of weight was achieved, compared with 10% in the study group (*p* = 0.619). Mean maximal BMI was 43.6 kg/m^2^; the initial BMI was 38.4 kg/m^2^. There were no surgical complications in that group. The LOS was 2.1 ± 0.5 day.

### Comparisons

The maximal weight did not differ significantly between ESKD and non-ESKD groups (128 ± 18.5 kg vs 131.5 ± 20.5 kg respectively, *p* = 0.583). The maximal BMI did not differ significantly (43.2 kg/m^2^ vs 43.6 kg/m^2^ respectively, *p* = 0.66). The change in BMI in the preparatory period was similar between the groups (4.36 vs 5.13 respectively, *p* = 0.6). The characteristics of follow-up measurements were presented in Table 2.

**Table 2:** Weight loss in ESKD and non-ESKD patients

	ESKD group	Non-ESKD group	*p*
Maximum BMI (mean/median)	43.2/43.5	43.6/41.5	0.663
Initial BMI (mean/median)	38.7/37.9	38.4/37.9	0.973
ΔBMI	4.36	5.13	0.608
Percentage BMI loss	10%	11%	0.640
%EBMIL	22%	25%	0.650
Follow-up visit 1 month after surgery (*n* = 20/20)			
BMI	33.8/33.5	34.3/34.2	0.883
ΔBMI	4.4	4.1	0.763
Percentage BMI loss	11%	10%	0.651
%EBMIL	33%	30%	0.586
Follow-up visit 3 months after surgery (*n* = 18/20)			
BMI	24.8	32/32	0.073
ΔBMI	8.4	6.5	0.077
Percentage BMI loss	22%	17%	0.038
%EBMIL	65%	49%	0.032
Follow-up visit 6 months after surgery (*n* = 18/18)			
BMI	22.8	29.4/29.4	0.151
ΔBMI	10.3	8.6	0.228
Percentage BMI loss	27%	22%	0.126
%EBMIL	79%	66%	0.100
Follow-up visit 12 months after surgery (*n* = 14/17)			
BMI	28.9	29.4/29.4	0.748
ΔBMI	12.3	9.4	0.275
Percentage BMI loss	25%	24%	0.716
%EBMIL	90%	68%	0.190

*n* = number of patients in ESKD and non-ESKD group respectively

**Fig. 1:**
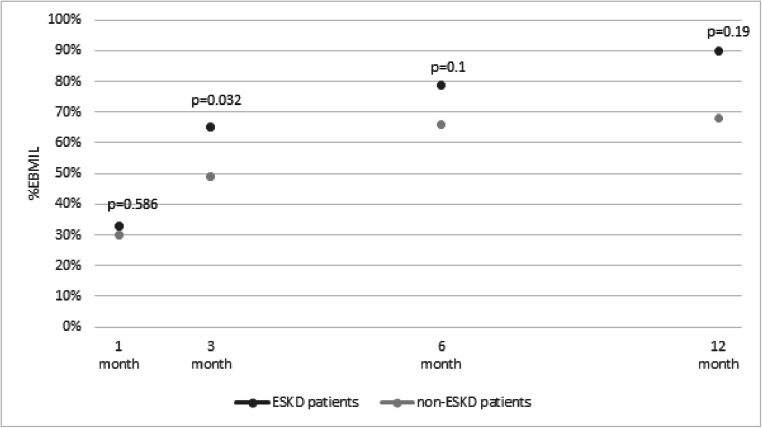
The comparison of the percentage of excess BMI loss after bariatric surgery between ESKD and non-ESKD patients (*p* < 0.05 statistically significant)

Bariatric surgery resulted in weight loss compared on four follow-up points. The comparison of %EBMIL in ESKD and non-ESKD patients is illustrated in Fig. 1.

## Discussion

In this study, we have demonstrated that ESKD patients can achieve substantial weight loss comparable with non-ESKD patients during surgical treatment of morbid obesity as preparation to kidney transplantation. This was to our knowledge never demonstrated in a matched pair analysis. The aim of this study was to evaluate weight loss in the preparatory period, and after bariatric surgery, in morbidly obese patients with ESKD before kidney transplantation, compared with non-ESKD morbidly obese patients.

In the literature, the most frequent cause for non-inclusion into the transplant waiting list is attributed to obesity (up to 30%) [12]. Based on the analysis of 19,524 dialysis patients, patients with BMI ≥40 kg/m^2^ were half as likely to undergo kidney transplantation than those with a BMI between 21 and 31 kg/m^2^ [5]. Several obesity treatments were proposed. Those including behavioral modification (physical activity, dietary modification) result only in a mean of 5 to 15% weight loss, and that result was maintained only in 5.3 to 10.5% of participants during a 1-year observation [13]. Similarly, psychosocial interventions such as group-based diet, group physical activity are related to a 3.5-kg weight loss in 6 months (95% CI, − 4.2 to − 2.8) and 3.4 kg in 12 months (95% CI, − 4.2 to − 2.9) [14]. Among all weight loss intervention, surgical treatment was considered the most effective. Our results present significant reduction of BMI, similar to matched pairs without ESKD in the 1-year follow-up (Table 2). In other studies, patients undergoing RYGB lost 29.9% (95% CI, 29.3–30.5%) more of their baseline weight at the 1 year follow-up than the non-surgical matches [15]. For LSG, 1-year weight loss was 23.4% (95% CI, 21.8–24.7%) [15]. As compared with behavioral modifications, reduction of BMI at 1 year was − 11.3 kg/m^2^ for OAGB, − 10.1 kg/m^2^ for sleeve gastrectomy (SG), and − 9.0 kg/m^2^ for RYGB [16]. The achieved weight loss was maintained for a long time (28.6% (95% CI, 19.5–37.6%) of their initial weight at 10 years) [15]. Those studies have shown that bariatric surgery not only can allow a 1-year substantial weight loss but also is the most effective way of achieving sustained weight reduction in obese patients [16]. The type of surgical procedure of choice in ESKD patients is still discussed. Our patients were qualified to a specific type of weight loss surgery according to the IFSO guidelines for the general population, which may in turn be inappropriate for ESKD patients [9]. Over the years, SG has replaced RYGB in that group of patients because of its relatively safe profile [17, 18]. Our national practice guidelines are not following those recent results [19, 20]. In morbidly obese patients with the indications for intestinal bypass methods, OAGB and RYGB were successfully implemented with the caution of hyperoxaluria presence during the rapid weight loss period and the risk of nephrolithiasis [21]. ESKD obese patients often fail to non-surgical intervention and are considered refractory to behavioral therapy. Bariatric surgery offers them not only a decrease in BMI but also an improvement of the altered kidney function and deceleration of the progression of renal diseases in patients with ESKD of various etiologies. Our matched pair analysis resulted in comparable weight loss in the preparatory period before bariatric surgery. Pretransplant renal replacement therapy of ESKD can influence the ability of patients to adhere to preoperative period weight loss instructions (i.e., steroids, osteoporosis) [22]. Despite those burdens, the results of the preparatory period did not differ significantly between the compared groups. They achieved − 4.36% and − 5.13% BMI loss in the preparatory period respectively, *p* = 0.608. The ESKD patients lost 21.78% of excess BMI and non-ESKD lost 24.53% EBMI. Moreover, our ESKD patients strictly meet the mandatory weight loss criteria (the median preoperative total weight change was − 10.2% and − 11.6% in the ESKD and non-ESKD groups respectively; *p* = 0.619) [23]. The preparatory weight reduction was not impaired by comorbidities (in our material, no statistical difference between groups except from hypertension). In the literature, a satisfactory preoperative weight loss correlates positively with postoperative weight loss as well as weight loss after 1 year [24, 25]. Moreover, successful preoperative weight loss is associated with a significant decrease in perioperative complications in a systematic review on 4611 patients (2 studies on 1234 patients) [25].

In our study, ESKD patients have comparable postoperative weight loss to non-ESKD obese patients (Fig. 1). The groups did not differ significantly in postoperative weight loss measured during the 1-year follow-up (1-year BMI was 28.9 kg/m^2^ vs 29.4 kg/m^2^, *p* = 0.748 and %EBMIL 90.5% vs 67.5%, *p* = 0.190 in ESKD and non-ESKD patients respectively). This fact indicates the possibility of safe and effective weight loss similar to non-ESKD. Although in the literature ESKD patients experienced a higher risk of postoperative complications compared with those without kidney disease, the absolute complication rates were low and bariatric treatment is considered safe in that group of patients [26]. Interestingly, in our study, the weight loss and decrease in BMI are significantly higher in the first month following bariatric surgery. This can be related to changes in plasma adipocytokines following surgery and a positive correlation with lowering insulin resistance in ESKD patients [27]. Moreover, ESKD patients in that period lost more excessive weight and the relative cardiovascular risk is starting to decline faster than non-ESKD patients. It was proven that weight loss is related to remission of diabetes (remission rate up to 61%), low-density lipoproteins (73%), and blood pressure (63%) [28, 29]. Not only weight loss but also resolution of ESKD-related comorbidities (diabetes mellitus, hypertension, hyperlipidemia, coronary heart disease, sleep apnea) is associated with metabolic and clinical benefits and lowers the risk of kidney transplantation.

According to the European Renal Best Practice Guideline recommendations, patients with a BMI > 30 kg/m^2^ should reduce weight before transplantation [19]. Literature data on the association of obesity and post-transplant graft function is conflicting. It was proven that obesity was associated with a higher risk of delayed graft function, graft loss, and lower patient and graft survival, but interestingly, obese individuals without comorbidities can experience similar survival to non-obese recipients [30–32]. However, often due to obesity-associated comorbidities, their access to the transplant list and transplantation is inferior to non-obese [5, 33]. Bariatric surgery is considered as a safe method of obesity treatment in that specific group of patients and feasible as a bridge therapy to kidney transplantation [6, 34–36]. Additionally, weight loss after bariatric surgery may improve kidney function to an acceptable level and delay the qualification to dialysis therapy in patients undergoing bariatric surgery before ESKD development. It was proven that weight loss resulted in an improvement of proteinuria and albuminuria and normalization of the glomerular filtration rate [22]. For dialyzed ESKD patients, bariatric surgery can slow the progression and allows them to stay on the transplant waiting list. Eight of our patients were already successfully transplanted within a few months up to 3 years after bariatric surgery. The optimal interval between the bariatric procedure and kidney transplantation has not been established yet. Long-term follow-up after bariatric surgery can be important in setting the optimal time of transplantation in obese ESKD patients. Furthermore, pretransplant weight loss as a result of bariatric surgery can improve the prehabilitation process before kidney transplantation, and as a result improve the functional and physiological capacity for a fast recovery sooner after kidney transplantation. Larger, long-term studies are needed to analyze the durability of this improvement and the effects on renal transplantation outcomes.

Our study has the following limitations. First, the results need to be interpreted acknowledging that the effects of surgery may vary, based on the characteristics of the individual patient, i.e., age, sex, and pre-surgery BMI comorbidities. Second, the group size was small, and the analysis was performed retrospectively. Although we matched patients for type of bariatric procedure performed, there are many factors related to specific procedure qualifications which may impact upon the results. Third, follow-up was limited to 1 year, thus long-term results of bariatric surgery in that group of patients were not analyzed. Lastly, three different bariatric procedures are included in analysis, which blurs the difference of outcome related to procedure type [37]. Research aimed to indicate the most effective type of bariatric surgery in ESKD patients is needed.

## Conclusion

Successful kidney transplantation requires elimination of possible pretransplant risks. Obesity is related to a higher risk of intra- and postoperative complications. Morbidly obese kidney transplantation candidates benefit from bariatric surgery and can be eagerly included in bariatric surgery weight loss programs. Bariatric surgery allows efficient pre-transplantation weight loss results, and the procedures in ESKD patients seem as safe as previously published.
